# Resource dependency and strategy in healthcare organizations during a time of scarce resources: evidence from the metropolitan area of cologne

**DOI:** 10.1108/JHOM-12-2020-0478

**Published:** 2021-07-07

**Authors:** Lena Ansmann, Vera Vennedey, Hendrik Ansgar Hillen, Stephanie Stock, Ludwig Kuntz, Holger Pfaff, Russell Mannion, Kira Isabel Hower, Cologne Research and Development Network (CoRe-Net) Study Group

**Affiliations:** Department of Health Services Research, School of Medicine and Health Sciences , University of Oldenburg , Oldenburg, Germany; Institute for Health Economics and Clinical Epidemiology, University Hospital Cologne , Cologne, Germany; Department of Business Administration and Health Care Management, University of Cologne , Cologne, Germany; Faculty of Human Sciences and Faculty of Medicine, Institute of Medical Sociology, Health Services Research, and Rehabilitation Science (IMVR), University of Cologne , Cologne, Germany; Health Services Management Centre, University of Birmingham , Birmingham, UK; Institute of Medical Sociology, Health Services Research, and Rehabilitation Science , University of Cologne , Cologne, Germany

**Keywords:** Resources, Shortage, Resource-dependency, Strategy, Organization, Healthcare, Environment, Pressure, Personnel development

## Abstract

**Purpose:**

Healthcare systems are under pressure to improve their performance, while at the same time facing severe resource constraints, particularly workforce shortages. By applying resource-dependency-theory (RDT), we explore how healthcare organizations in different settings perceive pressure arising from uncertain access to resources and examine organizational strategies they deploy to secure resources.

**Design/methodology/approach:**

A cross-sectional survey of key decision-makers in different healthcare settings in the metropolitan area of Cologne, Germany, on perceptions of pressure arising from the environment and respective strategies was conducted. For comparisons between settings radar charts, Kruskal–Wallis test and Fisher–Yates test were applied. Additionally, correlation analyses were conducted.

**Findings:**

A sample of *n* = 237(13%) key informants participated and reported high pressure caused by bureaucracy, time constraints and recruiting qualified staff. Hospitals, inpatient and outpatient nursing care organizations felt most pressurized. As suggested by RDT, organizations in highly pressurized settings deployed the most vociferous strategies to secure resources, particularly in relation to personnel development.

**Originality/value:**

This study is one of the few studies that focuses on the environment's impact on healthcare organizations across a variety of settings. RDT is a helpful theoretical foundation for understanding the environment's impact on organizational strategies. The substantial variations found between healthcare settings indicate that those settings potentially require specific strategies when seeking to address scarce resources and high demands. The results draw attention to the high level of pressure on healthcare organizations which presumably is passed down to managers, healthcare professionals, patients and relatives.

## Background

Modern healthcare systems are under increasing pressure to improve the quality and efficiency of the services they deliver ( OECD and European Union, 2016 , 2018 ) while at the same time facing severe resource constraints, particularly in relation to worsening workforce shortages. ( Scheffler and Arnold, 2019 ; World Health Organization, 2016 ). Healthcare organizations operating in such a resource constrained environment are constantly required to find a dynamic fit between their organization and the environment ( Moore, 2000 ). According to Scott “environments are all those significant elements outside the organization that influence its ability to survive and achieve its ends” ( Scott and Davis, 2007 , p. 19). The environment can thus be regarded as a store of resources as well as a potential source of opportunities and constraints, demands and threats. Healthcare organizations in different healthcare settings (e.g. hospitals, nursing homes, general practitioners) may operate in very different environments and thus may require different strategies in order to find a strategic fit. This article examines how healthcare organizations in different healthcare settings in Germany perceive pressure due to limited and uncertain resources within their environment and explores the range of organizational strategies they deploy to secure such resources.

### Resource-dependency in healthcare organizations

Healthcare organizations – like all organizations – need to adapt to their specific physical, technological, cultural, and social environments and interact within broader systems ( Scott and Davis, 2007 ). For healthcare organizations, the broader system comprises first and foremost the healthcare system, but also the broader economic and science systems. Resource-dependency-theory (RDT) is one of the most influential organizational theories for examining how organizational environments shape organizational strategies to access resources. ( Pfeffer and Salancik, 1978 ). Pfeffer and Salancik from an economic perspective maintain that in times of environmental uncertainty arising from scarce resources, constant changes and complex interrelations between organizations, decision-makers in organizations deploy a range of strategies to secure resources and to reduce uncertainty caused by a changing and uncertain environment ( Pfeffer and Salancik, 1978 ). It can be assumed that environmental uncertainties and interdependencies between organizations result in perceived pressure on healthcare organizations to adapt and align their strategies to the environment ( Pettigrew *et al.* , 1992 ). From this perspective, environmental uncertainty is assumed to influence organizational strategies which in turn ultimately influence organizational performance.

RDT has previously been applied to the healthcare sector ( Yeager *et al.* , 2014 ). The environment of healthcare organizations in many countries including Germany is increasingly characterized by uncertainty. First, the availability of and access to resources (defined as *munificence* in the RDT) is limited. Workforce resources are perceived as a major problem within the German health care system as healthcare organizations are unable to fill vacancies with qualified personnel ( Scheffler and Arnold, 2019 ). At the same time, demographic changes are increasing demand on scarce healthcare resources. Moreover, in Germany and many other countries, healthcare reforms have led to changes in funding and access to services ( Simonet, 2010 ; Mattei *et al.* , 2013 ). Second, since the German health care system is regularly subject to reform, frequent changes within the environment ( *dynamism* ) occur ( Ozegowski and Sundmacher, 2012 ). These constant changes shape the demands that healthcare organizations are required to meet and thereby define the resources used. Third, interrelations between organizations ( *complexity* ) within the German healthcare system become more complex. This is due to efforts to increase efficiency and patient value ( Simonet, 2010 ) by fostering cross-sectoral cooperation, e.g. between hospitals and ambulatory care. Thus, healthcare organizations are increasingly constrained by a network of interdependencies between healthcare organizations, whose uncertain actions increase the healthcare organizations' general uncertainty ( Ozegowski and Sundmacher, 2012 ).

According to RDT, when healthcare organizations perceive the environment to be uncertain, they direct organizational strategies towards securing additional resources ( Pfeffer and Salancik, 1978 ). However, there have only been a limited number of studies from a RDT perspective exploring the association between the environment within which healthcare organizations operate and their respective organizational strategies ( Yeager *et al.* , 2014 ). None have focused on exploring these issues within the German healthcare context. Besides the tendency of healthcare organizations in Germany to extend their services and volume in order to gain resources and thereby overproduce healthcare services ( Grote Westrick *et al.* , 2019 ), it is largely unknown how they manage the ever-present scarcity of resources. Moreover, in health services research generally very few studies investigate similarities and differences between healthcare organizations from different healthcare settings, but mostly compare healthcare within a defined setting. These settings can range from general practitioner (GP) practices to hospitals and from ambulatory nursing care to nursing homes. Healthcare organizations in these settings vary in their structures and processes and presumably in their strategies for accessing external resources. One example could be the varying availability of qualified healthcare staff in different healthcare settings, which can lead to more or less uncertainty in terms of staff resources and in turn to different strategies for staff recruitment. Thus, comparing uncertainties and strategies between healthcare settings could provide insights into setting-specific associations. To the best of our knowledge, differences in the perception and management of resources among healthcare organizations across different settings have not previously been studied in any detail ( Yeager *et al.* , 2014 ). This study aimed to fill this gap in knowledge and evidence.

### Healthcare organizations in Germany

Compared to many other healthcare systems in the EU, Germany has a very strong ambulatory care sector, which is clearly separated from the inpatient care sector. Physicians offer not only primary care in solo or joint private practices, but almost all specialties are represented in ambulatory care ( Blümel and Busse, 2017 ). Similarly, therapists predominantly provide care in private practices. The hospital sector is dominated by public hospitals, while private for-profit hospitals have increased in recent years ( Heimeshoff *et al.* , 2014 ). Individuals on the whole exercise free choice over GPs, specialists, and, if referred to inpatient care, hospitals. Medical rehabilitation takes place in inpatient or outpatient rehabilitation clinics or centers. Long-term care is provided in inpatient nursing homes and by ambulatory nursing services for people who are cared for at home. The majority of German citizens are covered by statutory health insurance, but around 11% of the population – mostly those with a high income – are insured privately ( Statistisches Bundesamt, 2019 ).

### Research aim

This study aims to address the following research questions using the RDT as an organizing framework: How do healthcare organizations in different settings perceive environmental pressure due to the *uncertainty of resources* ? What range of *organizational strategies* do they pursue in order to secure resources? Is perceived environmental pressure associated with organizational strategies as suggested by RDT? It is hoped that the findings of this research will contribute to a better understanding of how healthcare organizations in different settings deploy strategies to mitigate an uncertain and resource constrained environment.

## Methods

### Study design, recruitment and data collection

Data was collected as part of the research project OrgValue (Characteristics of value-based health and social care from organizations‘ perspectives; German Registry for Clinical Trials - DRKS00011925), which is part of the CoRe-Net (Cologne Research and Development Network) – a network of researchers and healthcare providers collaborating to redesign healthcare in an urban region ( Karbach *et al.* , 2017 ). The city of Cologne, situated in the mid-west of Germany, has a population of more than one million inhabitants and is the fourth largest city in Germany. Cologne is an urban core of a large and densely populated metropolitan area in the Midwest of Germany. As the urban core it has strong ties with surrounding rural areas and cities and thus has a large catchment area in terms of specialist healthcare. Thereby, it represents the urban core of a typical European metropolitan area in densely populated regions. OrgValue is a cross-sectional study examining healthcare organizations in the city of Cologne with respect to the implementation of patient-centered and resource-oriented healthcare ( Ansmann *et al.* , 2018 ). Whereas OrgValue encompasses both qualitative and quantitative data collection, the focus of this article is on the quantitative arm of the study. Data were collected by a standardized postal survey of key decision makers in healthcare organizations in Cologne. Decision-makers were selected from clinicians or managers in leading positions with decision-making authority and leadership responsibility ( Lavrakas, 2008 ). A key informant survey was chosen because it enables a substantially larger number of organizations to be surveyed at lower cost ( Kumar *et al.* , 1993 ). The person or steering board in the highest position within each organization was contacted by post with study information accompanied by an informed consent form, the questionnaire and prepaid return envelopes. Poor German language skills was an exclusion criterion. The included healthcare organizations comprised (1) inpatient nursing homes and hospices, (2) hospitals, (3) inpatient and outpatient rehabilitation organizations, (4) physician practices (GPs and cardiology specialists), (5) outpatient nursing and hospice care organizations, and (6) psychotherapy practices. Recruitment took place based on the contact information gathered from the Association of Statutory Health Insurance Physicians North Rhine and web-based search. The survey was conducted according to Dillman's Total Design Method with two personalized reminders sent out to non-responders ( Dillman, 1978 ). In addition, several strategies shown to increase survey response rates were used (e.g. personalized letters, highlighting the academic origin, prepaid envelopes, promoting the study through outreach activities). As a financial incentive, a donation of 1€ per completed questionnaire was sent to a charity organization for disadvantaged children in the city of Cologne, survey feedback via anonymous benchmarking reports ( Ivers *et al.* , 2012 ), and outreach events for organizational learning were promoted. This study received ethical approval from the Ethics Committee of the Medical Faculty of the University of Cologne (reference nr. 17–210). Written informed consent was obtained from all study participants.

### Instruments

Within the OrgValue research project, qualitative interviews with decision-makers on the implementation of patient-centeredness and resource-orientation in healthcare organizations had already been conducted prior to the survey. The results of the interviews have previously been published ( Hower *et al.* , 2019 ) and informed the survey development by identifying relevant aspects and determinants of healthcare organizations' strategies in order to secure resources and ensure patient-centeredness. The selection of validated instruments was also guided by international frameworks on organizational determinants of patient-centeredness ( Shaller, 2007 ; Luxford *et al.* , 2011 ; Scholl *et al.* , 2014 ; Institute of Medicine, 2001 ) and implementation research ( Damschroder *et al.* , 2009 ). Instruments on resource orientation used for this analysis are in part adapted from existing instruments and in part self-developed due to a lack of validated instruments with a specific focus on the healthcare setting. Six cognitive pretest interviews using the think-aloud technique ( Beatty and Willis, 2007 ) were conducted to assure comprehension, practicability and completeness. All survey items described below are shown in an additional table ( supplementary Table 1 ). None of the survey instruments used are under license.

#### Perceived environmental pressure

We assume that environmental uncertainties result in perceived pressure on healthcare organizations to adapt to such uncertainties ( Pettigrew *et al.* , 1992 ). Based on the interview results, the items were adapted from a previous instrument developed for a key informant survey in breast cancer centers ( Pfaff *et al.* , 2012 ). Five items were used to measure the decision-makers' perception of pressures stemming from the organizations' environment: pressure for change due to competition, economic pressure, pressure for recruitment of qualified staff (example item “Our organization is under pressure to recruit qualified staff”), time pressure and pressure by bureaucracy. The items were scored on a five-point Likert scale in two steps. First, participants were asked to agree or disagree to the item and second, those who agreed were asked to evaluate to what extent the organization is distressed by the experience stated (ranging from “not distressed” to “very largely distressed”).

#### Organizational strategies

We assume that the perceived environmental pressure leads to organizational strategies to secure additional organizational resources. The survey contained various single items on organizational strategies with regard to personnel development, quality management, external staff and outsourcing. In regard to personnel development five single items assessed organizational support for staff trainings and continuing education in terms of (1) motivating staff, (2) regarding trainings and continuing education as working hours, (3) covering fees, (4) covering travelling and accommodation costs as well as (5) offering inhouse or nearby trainings. The items were preceded by the question “Does your organization support the participation in staff trainings by the following measures?” and were answered on a binary scale (“yes” or “no”). Further strategies for personnel development regarding occupational health promotion and supervision for the healthcare workforce were assessed with two single items (“Our organization enables measures for occupational health promotion for staff (e.g. cooperation with gyms, courses on stress management)” and “In our organization supervision for the staff is carried out”). Both items were answered along a four-point Likert scale ranging from “do not agree at all” to “completely agree”. The organizational strategy towards quality management was assessed with one item (“Our organization has a uniform quality management”) to be answered on a four-point Likert scale ranging from “do not agree at all” to “completely agree”. Organizational strategies towards the employment of external staff and outsourcing were assessed by two items (“Has external staff (honorary staff, temporary employees) been employed in your organization within the last 12 months?” and “Have parts of your organization been outsourced?”). Both items had to be answered on a binary scale (“yes” or “no”).

### Statistical analysis

Descriptive statistics were used to characterize the participants and their respective organizations. To display differences between healthcare organizations from different settings the results were analyzed descriptively. Radar charts were used to display responses by healthcare settings. Due to the limited and heterogeneous sample sizes across healthcare settings, the Kruskal–Wallis test was used to test for statistically significant differences between the healthcare settings. The Kruskal–Wallis test is an alternative to variance analysis, when sample sizes are small and when ordinal measures are used. In regard to nominal measures with small sample sizes, the Fisher–Yates test was applied. To test correlations between environmental pressure and organizational strategies Cramer's V and Spearman's rank correlation coefficient were calculated. The significance level for all analyses was 0.05. IBM SPSS Statistics 24 has been used for the statistical analyses.

## Results

From *n* = 1790 healthcare organizations contacted, *n* = 237 (13%) returned completed questionnaires. The variety of healthcare organizations contacted and their response rate by healthcare setting is shown in Table 1 . Whereas the number of healthcare organizations eligible for the study in Cologne is highest for physician practices and psychotherapy practices, the number of large inpatient healthcare providers, such as hospitals and rehabilitation organizations, is much smaller. Thus, the sample is naturally dominated by many small outpatient healthcare organizations. The response rates however vary between healthcare settings, with rehabilitation organizations and hospitals having response rates between 46 and 63%, while inpatient nursing homes or hospices as well as physician practices and psychotherapy practices have a lower response rate of about 12%.

The majority of decision-makers were between 46 and 65 years old and about 70% were female (see Table 2 ). The gender distribution varied from 53.8% participating female decision-makers in hospitals to 79.2% in psychotherapy practices. Most participants had a medical, psychology or nursing background, but the distribution varied strongly between healthcare settings. Most of the participants worked in their organization as a therapist (45.0%) or physician (27.3%). Whereas 16.2% worked in management or administration. The majority (86.4%) of the participants are currently working directly with patients. The size of healthcare organizations assessed by the number of employees and patient volume per day show a wide variation between healthcare settings.

### Perceived environmental pressures

**Figure 1:**
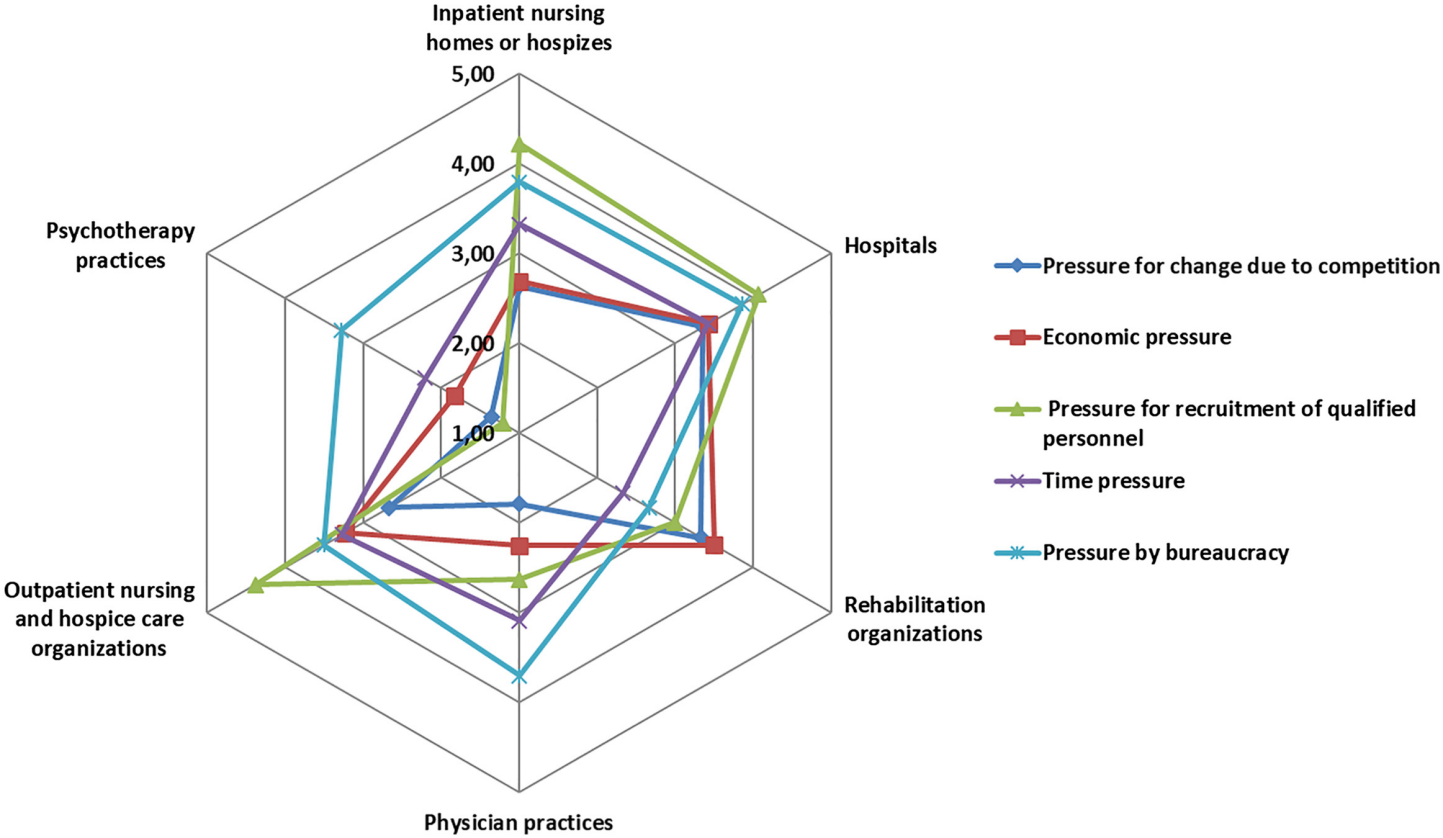
Radar chart of perceived environmental pressures by healthcare setting (1 = not present, 5 = very distressed)

Figure 1 displays the healthcare organizations' perceived environmental pressures on a scale from 1 (not present) to 5 (very distressed). Healthcare organizations in general rated a mean of 3.5 (SD 1.0) for pressure by bureaucracy, followed by 2.8 (SD 1.3) for time pressure, 2.4 (SD 1.5) for pressure for recruitment of qualified personnel, 2.3 (SD 1.2) for economic pressure, and 2.0 (SD 1.1) for perceived pressure for change due to competition. The radar chart reveals profiles of perceived environmental pressure varying between healthcare settings. The Kruskal–Wallis test reveals that these differences between healthcare settings are significant in all types of perceived pressure ( *p* < 0.01) (detailed results for Kruskal–Wallis tests are not shown here). Decision-makers in hospitals reported that they were highly pressurized in almost all areas, with the highest scores relating to recruitment of qualified personnel. Inpatient nursing homes or hospices reported even higher pressures with regard to the recruitment of qualified personnel and moderate to high scores for pressure by bureaucracy and time pressure. Rehabilitation organizations reported moderate to high economic pressure and pressure for change due to competition. Physician practices perceived a high pressure by bureaucracy, followed by a moderate to high time pressure. Outpatient nursing and hospice care organizations reported a very high distress due to pressure to recruit qualified staff. Psychotherapy practices reported a moderate to high pressure by bureaucracy, but all other types of pressure are perceived as moderate to low.

### Organizational strategies

**Figure 2:**
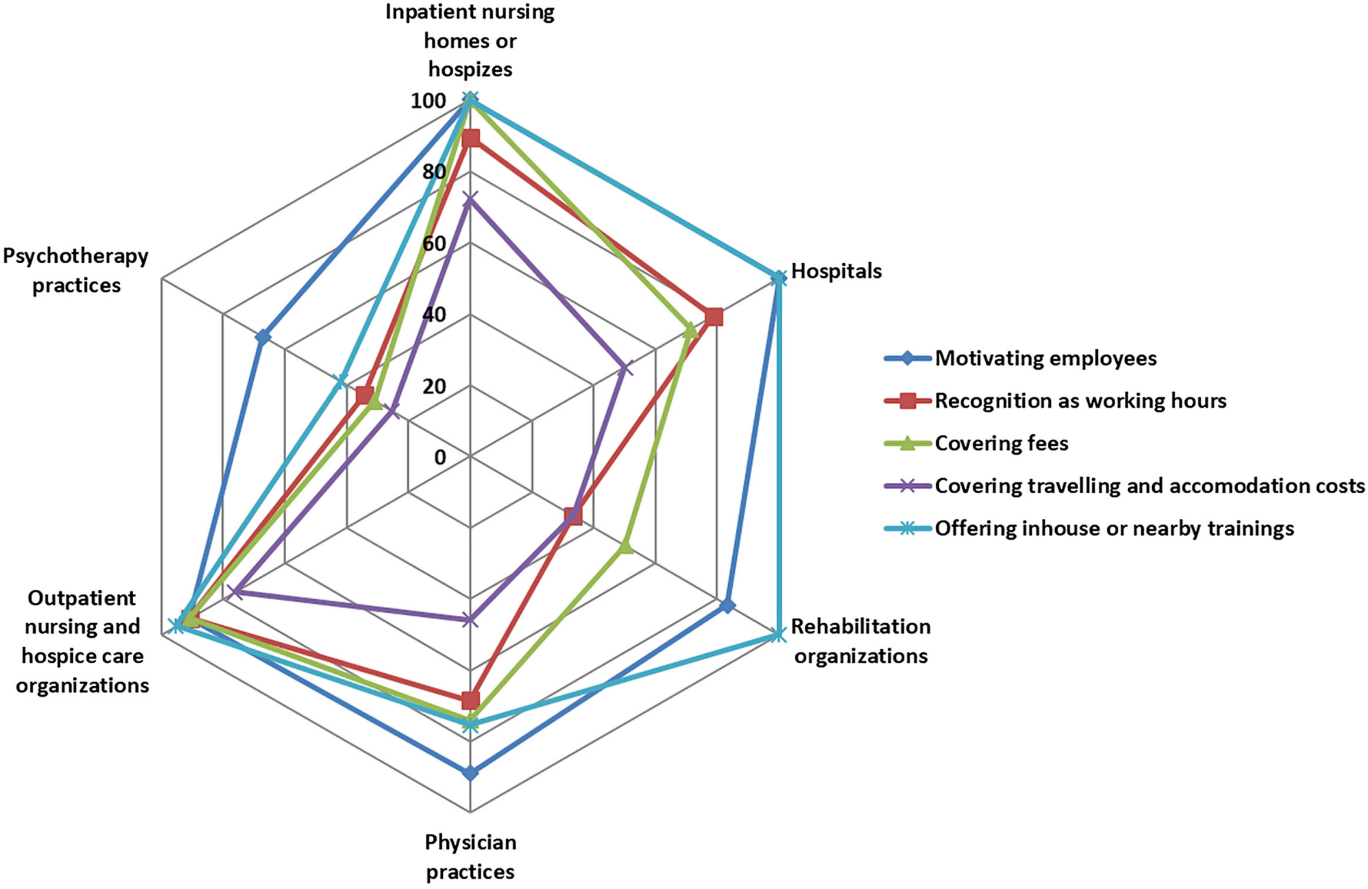
Radar chart of organizational strategies in regard to staff training and continuing education by healthcare setting in %

Of all healthcare organizations, the vast majority, 82.9% (SD 37.8) reported that they actively motivate staff to participate in staff training and continuing education. 60.8% (SD 49.0) reported regarding staff training and continuing education as working hours, 62.4% (48.5) reported to cover fees, 44.1% (SD 49.8) reported to cover travelling and accommodation costs and 70.1% (SD 45.9) reported to offer inhouse or nearby trainings. The Fisher–Yates test reveals significant differences by healthcare settings in all of these strategies ( *p* < 0.01). Figure 2 shows that inpatient and outpatient nursing and hospice care organizations – which also perceived the highest pressure for recruitment ( Figure 1 ) - reported a very strong strategy to support participation in staff training and continuing education. Hospitals, rehabilitation organizations and physician practices do partly support staff in participating in staff training and continuing education, but less often cover the costs. Also, physician practices compared to most other healthcare settings do less frequently offer inhouse or nearby trainings. Psychotherapy practices reported weak organizational strategies regarding staff training and continuous education.

**Figure 3:**
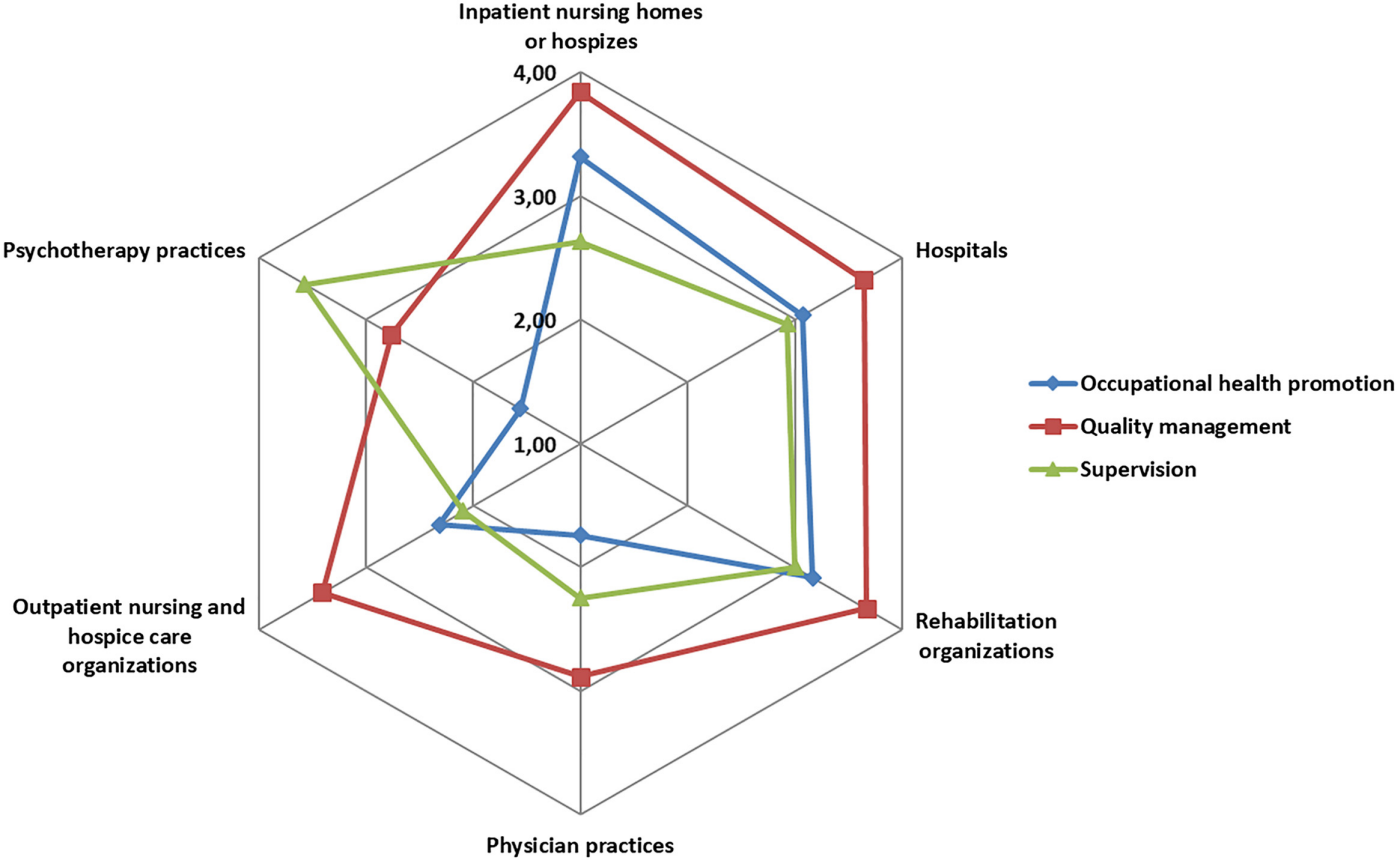
Radar chart of organizational strategies in regard to occupational health promotion, supervision and quality management by healthcare setting, 1 = do not agree at all, 4 = completely agree

With regard to the implementation of occupational health promotion, the mean score across all healthcare organizations was 2.0 (SD 1.1) on a scale ranging from 1–4. The mean for implementing supervision of staff was 2.9 (SD 1.2) and for implementing a comprehensive quality management 3.0 (SD 1.0). The Fisher–Yates test reveals significant differences by healthcare settings in all three of these strategies ( *p* < 0.01). Figure 3 shows that occupational health promotion is implemented more in inpatient nursing homes or hospices, hospitals and rehabilitation organizations compared to outpatient healthcare organizations. Psychotherapy practices report a high implementation of supervision for staff, compared to all other healthcare settings. Comprehensive quality management is reported to be highly implemented in all healthcare settings, except in psychotherapy and physician practices, where the degree of implementation is moderate to high.

**Figure 4:**
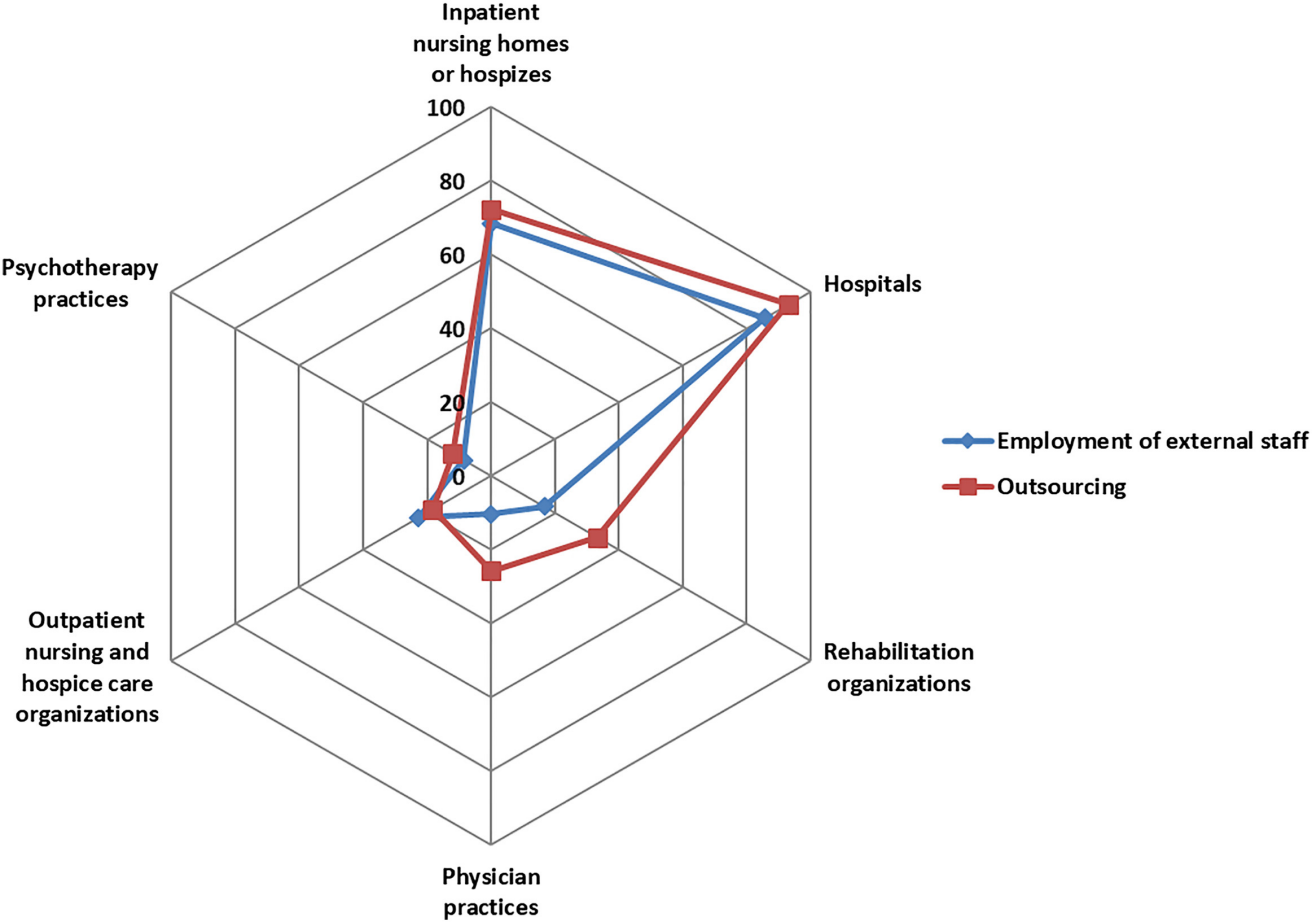
Radar chart of organizational strategies in regard to employment of external staff and outsourcing by healthcare setting in %

From all participating healthcare organizations, 20.2% employed external staff within the last 12 months (see Figure 4 ). In addition, 27.4% are outsourcing organizational units, such as laboratory and cleaning services. The Fisher–Yates test reveals significant differences by healthcare settings in both strategies ( *p* < 0.01). Whereas the majority (between 68.4 and 92.9%) of inpatient nursing homes or hospices as well as hospitals - which at the same time perceived the highest pressure to recruit qualified personnel ( Figure 1 ) - utilize external staff and outsourcing, in all other healthcare settings a minority (between 8.5 and 33.3%) are using it.

### Correlations between perceived environmental pressure and organizational strategies

To test the RDT's hypothesis that uncertainties in the organization's environment impact organizational strategies towards securing resources, correlation analyses between environmental pressure and organizational strategies were conducted (see Table 3 ). All types of environmental pressures were significantly positively correlated with various organizational strategies applied, confirming the hypothesis. Pressure for recruitment of qualified personnel showed significant correlations with all of the strategies studied. Correlations to all organizational strategies except staff supervision had a positive direction. Overall, weak to moderate correlations were found.

## Discussion

This study has examined how healthcare organizations perceive pressure arising from uncertain access to scarce resources within their environment and the range of organizational strategies they deploy to secure access to such resources. The key informant survey revealed that healthcare organizations in Cologne perceived high environmental pressure, particularly in terms of bureaucracy, time, and the recruitment of qualified staff, which reflect the uncertainty of resources described in the RDT. This corresponds with current evidence and ongoing debates in many healthcare systems around the need to reduce bureaucracy to facilitate more time spent on patient care ( Siegler *et al.* , 2015 ; Oxentenko *et al.* , 2010 ; Cunningham *et al.* , 2012 ) and increasing satisfaction in the workforce ( Cherry *et al.* , 2007 ). The findings also align with those on time constraints in medical care in different healthcare systems including Germany ( Konrad *et al.* , 2010 ). In addition, the problems associated with the recruitment of qualified staff are well-documented and are currently an urgent problem in many healthcare systems, including Germany ( Schermuly *et al.* , 2015 ; Scheffler and Arnold, 2019 ; Simeons *et al.* , 2005 ), as vacancies increasingly remain unfilled. The comparison across healthcare settings reveals substantial differences. Inpatient and outpatient nursing care organizations as well as hospitals, reported particularly high pressures, especially in terms of bureaucracy and recruitment. This indicates that hospitals as well as inpatient and outpatient nursing care organizations may carry a particularly high burden in terms of bureaucratic load in addition to their tasks in patient care. The burden of recruiting qualified staff underlines the current high workforce shortages in the German healthcare system, especially in hospital physicians and nurses across various healthcare settings ( Kasch *et al.* , 2016 ; Zander and Busse, 2017 ).

RDT assumes that during a time of insecure resources, which in this study is characterized for example by the shortage of qualified staff and time constraints, organizations adapt their strategies accordingly in order to secure resources ( Pfeffer and Salancik, 1978 ). Our results align in part with this assumption. Across all healthcare organizations studied, correlations between environmental pressure and organizational strategies applied were found to be significant. Correlations were particularly consistent regarding pressure to recruit qualified personnel. Inpatient and outpatient nursing care organizations as well as hospitals, which generally perceived the highest insecurity of resources, at the same time also devoted the highest effort into personnel development. Thus, the insecurity of resources in their environment, particularly shortage of qualified staff, possibly shaped the organizations' strategy to invest in retaining and qualifying their workforce by facilitating their participation in training and continuing education.

Occupational health promotion may also be an effective means for securing personnel resources by investing in healthcare professionals' well-being and resilience and thereby maintaining and strengthening their performance and work ability throughout their career ( Groene and Jorgensen, 2005 ). The physical and psychological burden on healthcare workers is one of the main reasons for nurses and physicians leaving their job ( Cherry *et al.* , 2007 ; Degen *et al.* , 2015 ). In our study, inpatient settings reported having implemented occupational health promotion the most, which may also be explained by the size of the organizations, since studies outside the healthcare sector showed that the implementation of occupational health promotion is strongly correlated with the organizations' size ( Ansmann *et al.* , 2012 ). Thus, occupational health promotion may be less determined by the organizations' perception of insecure personnel resources than by their capacities in terms of infrastructure and investments. Supervision for healthcare workers has to a great degree been implemented in psychotherapy practices, which was to be expected, since in psychotherapy, supervision is an established quality assurance and personnel development measure. Among all other organizations, supervision does not seem to be a key strategy for securing resources. Surprisingly, correlations between environmental pressure and supervision were consistently negative, indicating that increased uncertainty is associated with organizations rather deciding not to prioritize supervision of staff as an organizational strategy. On the other hand, comprehensive quality management systems have been implemented in most healthcare settings due to standards and requirements from the healthcare system. However, it is interesting to note that psychotherapy and physician practices reported a somewhat lower degree of implementation, which might be explained by their small organizational size.

In times of severe workforce shortages and a dynamic environment, healthcare organizations can try to compensate by employing external staff in non-permanent (sub) contracts for specific tasks such as fee-based physicians. In addition, healthcare organizations are able to outsource services and thereby transfer the responsibility for assuring the resources needed for these services to external contractors ( Machado Guimarães and Crespo de Carvalho, 2013 ). Both strategies are rarely used by hospitals and inpatient nursing care organizations, who together with outpatient nursing care organizations also reported the highest pressure to recruit qualified personnel. Again, aligning with RDT, our data showed that those organizations with high insecurity in terms of personnel resources made most use of external staff and outsourcing. Possibly, the size of organizations may play a role here as well, since outpatient nursing care organizations, which also reported high pressure in terms of recruitment, did not make use of the same strategies.

### Strengths and limitations

The results should be interpreted in the light of the study's strengths and limitations. Since most studies in the healthcare sector are limited to only one healthcare setting – mostly hospitals or GP practices – comparing a large variety of healthcare settings is a strength, since it can provide new and original insights ( Yeager *et al.* , 2014 ). Moreover, this is one of the very few studies applying the RDT as a theoretical approach in health services research. The response rate of 13% might imply a selection bias in favor of healthcare organizations with an interest in the areas covered in the study or with lower perceived environmental pressure. A comparison of responding and non-responding healthcare organizations in terms of characteristics such as size was not possible due to the lack of accessible statistics. For surveys of key informants in organizations a mean response rate of only 35% published in 2008 in academic journals in the management and behavioral sciences was found ( Baruch and Holtom, 2008 ). Within recent decades, response rates in organizational surveys are known to have decreased ( Swanson and Holton, 2005 ). However, Dillman's strategy ( Dillman, 1978 ) to increase response rates including two personal reminders and considering respective design and layout aspects have comprehensively been applied. Moreover, only few large healthcare organizations were included, since the study is focusing on only one large city, which naturally limits the number of hospitals, nursing homes and rehabilitation centers available. Nevertheless, this study allowed to map the health services landscape of Cologne, which is representative of many metropolitan areas in Germany and Europe. This enabled us to uniquely compare healthcare organizations in a shared environment. Unfortunately, it remains unknown whether participating organizations belong to the same umbrella organization or share common structures, which could lead to similarities in the investigated strategies. Of course, the small numbers limit the analytical methods that can be deployed. As this study focused on the German healthcare system, the results may not be directly transferable to other healthcare systems.

## Conclusion

Within health services research, few previous studies have focused on the impact of the healthcare organizations' environment across a variety of healthcare settings and this study has helped to fill this gap in knowledge. Future research should engage in these comparisons on a larger scale with higher numbers of healthcare organizations and possibly larger or multiple regions to be able to confirm the findings. It is clear that RDT theory is a valid and helpful theoretical foundation for helping to understand how an organization's environment influences organizational strategies and performance in healthcare, which hitherto has rarely been explored using this framework ( Yeager *et al.* , 2014 ). To comprehensively study the environments' impact using RDT, future studies should include performance data such as quality indicators and outcome metrics. The findings of this study can be used to raise awareness of the substantial differences that exist between healthcare settings. Organizations' environments and strategies seem to vary substantially between healthcare settings and thus they potentially require tailored strategies for coping with insecurities in their environment. In general, the results draw attention to the high level of pressure placed on health care organizations which presumably is relayed down to managers, healthcare professionals, patients, and relatives and should be a concern when designing healthcare systems and managing healthcare organizations.

## Figures and Tables

**Table 1:** Healthcare organizations contacted and response rates by healthcare setting

	Contacted	Completed	Response rate in %	Proportion of total response in %
Inpatient nursing homes or hospices	177	22	12.4	9.3
Hospitals	22	14	63.0	5.9
Rehabilitation organizations	13	6	46.2	2.5
Physician practices	665	80	12.0	33.8
Outpatient nursing and hospice care organizations	86	19	22.1	8.0
Psychotherapy practices	807	96	11.9	40.5
*Total*	*1790*	*237*	*13.2*	*100.0*

**Table 2:** Descriptives of the sample of decision-makers in healthcare organizations (no missing data present)

DECISION-MAKERS		Inpatient nursing homes and hospices		Hospitals		Rehabilitation organizations		Physician practices		Outpatient nursing and hospice care		Psycho-therapy practices		Total	
		*n*	%	*n*	%	*n*	%	*n*	%	*n*	%	*n*	%	*n*	%
Age	26–35	1	5.3	0	0.0	0	0.0	2	2.5	1	4.5	5	5.2	9	3.8
	36–45	2	10.5	1	7.7	0	0.0	12	15.0	5	22.7	24	25.0	44	18.6
	46–55	11	57.9	6	46.2	4	66.7	30	37.5	8	36.4	23	24.0	82	34.7
	56–65	5	26.3	6	46.2	2	33.3	29	36.3	7	31.8	33	34.4	82	34.7
	>65	0	0.0	0	0.0	0	0.0	7	8.8	1	4.5	11	11.5	19	8.1
Sex	Female	12	63.2	7	53.8	4	66.7	50	62.5	17	77.3	76	79.2	166	70.3
	Male	7	0.0	6	46.2	2	33.3	30	37.5	5	22.7	20	20.8	70	29.7
Background (multiple answers)	Medicine	0	0.0	7	53.8	4	66.7	61	76.3	0	0.0	4	4.0	76	30.8
	Psychology	1	4.0	0	0.0	0	0.0	15	18.8	0	0.0	92	92.0	108	43.7
	Nursing	15	60.0	6	46.2	0	0.0	0	0.0	15	65.2	1	1.0	37	15.0
	Social work	2	8.0	0	0.0	1	16.7	1	1.3	2	8.7	1	1.0	7	2.8
	Economics and finances	5	20.0	0	0.0	0	0.0	2	2.5	4	17.4	1	1.0	12	4.9
	Other	2	8.0	0	0.0	1	16.7	1	1.3	2	8.7	1	1.0	7	2.8
Occupation (multiple answers)	Nurse	7	28.0	4	36.4	0	0.0	0	0.0	11	32.4	0	0.0	22	8.5
	Physician	1	4.0	7	63.6	4	57.1	57	68.7	0	0.0	2	2.1	71	27.3
	Therapist	1	4.0	0	0.0	2	28.6	19	22.9	2	5.9	93	95.9	117	45.0
	Management and administration	16	64.0	0	0.0	0	0.0	6	7.2	16	47.1	1	1.0	42	16.2
	Other	0	0.0	0	0.0	1	14.3	1	1.2	5	14.7	1	1.0	8	3.1
Are you working in patient care?	Yes	4	21.1	7	53.8	5	83.3	78	97.5	14	63.6	96	100.0	204	86.4
	No	15	78.9	6	46.2	1	16.7	2	2.5	8	36.4	0	0.0	32	13.6
Healthcare organizations		mean	SD	mean	SD	mean	SD	mean	SD	mean	SD	mean	SD	mean	SD
Number of employees		89.6	40.5	448.5	274.2	31.0	15.8	11.2	25.4	36.6	36.5	6.2	32.0	43.3	122.0
Patient volume per day		99.8	50.6	330.5	289.0	88.8	81.9	73.7	59.4	119.7	87.2	19.1	87.2	73.6	122.5
*Total*		*22*		*14*		*6*		*80*		*19*		*86*		*237*	

**Table 3:** Results of correlation analysis between environmental pressure and organizational strategies

	Organizational strategies																			
	Organizational support for staff trainings and continuing education										Occupational health promotion^b^		Quality management^b^		Staff supervision^b^		External staff^a^		Outsourcing^a^	
Environmental pressure	Motivating employees^a^		Recognition as working hours^a^		Covering fees^a^		Covering travelling and accommodation costs^a^		Offering inhouse or nearby trainings^a^											
	*r*	*p*	*r*	*p*	*r*	*p*	*r*	*p*	*r*	*p*	*r*	*p*	*r*	*p*	*r*	*p*	*r*	*p*	*r*	*p*
Pressure for change due to competition	*0.218*	*0.041*	*0.226*	*0.035*	*0.228*	*0.031*	0.184	0.146	*0.299*	*0.001*	*0.278*	*<0.001*	*0.254*	*<0.001*	*−0.259*	*<0.001*	*0.321*	*<0.001*	*0.347*	*<0.001*
Economic pressure	*0.229*	*0.026*	0.195	0.102	*0.241*	*0.018*	0.131	0.484	*0.310*	*0.001*	*0.222*	*0.001*	*0.181*	*0.006*	*−0.352*	*<0.001*	*0.259*	*0.004*	*0.275*	*0.002*
Pressure for recruitment of qualified personnel	*0.404*	*<0.001*	*0.426*	*<0.001*	*0.483*	*<0.001*	*0.304*	*0.001*	*0.606*	*<0.001*	*0.384*	*<0.001*	*0.316*	*<0.001*	*−0.477*	*<0.001*	*0.405*	*<0.001*	*0.348*	*<0.001*
Time pressure	*0.242*	*0.015*	0.158	0.280	0.184	0.141	0.087	0.821	*0.240*	*0.018*	*0.139*	*0.040*	0.111	0.093	*−0.334*	*<0.001*	*0.205*	*0.044*	*0.282*	*0.001*
Pressure by bureaucracy	0.201	0.074	*0.217*	*0.047*	0.152	0.319	*0.240*	*0.020*	*0.260*	*0.007*	0.028	0.678	0.019	0.779	*−0.243*	*<0.001*	0.197	0.061	*0.222*	*0.026*

**Note(s):** ^a^Cramer's V

^b^Spearman's rank correlation coefficient; significance level *p* < 0.05 (marked in italic)

## Appendix

The supplementary file is available online for this article.
