# Supplementary material for: Resource dependency and strategy in healthcare organizations during a time of scarce resources: evidence from the metropolitan area of cologne
Source: J Health Organ Manag. 2021 Jul 7;35(9):211–27. doi: 10.1108/JHOM-12-2020-0478 (PMC9136866; doi:10.1108/JHOM-12-2020-0478)
Supplement: Supplementary file 1 [file jhealthorganmanag-35-0211.docx]

| **Items** | **Response options** |
| --- | --- |
| Perceived environmental pressure | |
| (1) Our organization competes with other organizations and therefore underlies pressure for change. | 1=no, 2=yes, not distressed, 3=yes, somewhat distressed, 4=very distressed, 5=yes, very largely distressed |
| (2) Our organization is under pressure to be in the black. |  |
| (3) Our organization is under pressure to recruit qualified staff. |  |
| (4) Our organization is under pressure to complete tasks in a short time. |  |
| (5) Our organization is under pressure to document things. |  |
| Organizational strategies: Personnel development | |
| *Does your organization support the participation in staff trainings by the following measures?* | |
| (1) Actively addressing and motivating staff to participate in trainings and continuing education | 0=no, 1=yes |
| (2) Full recognition of trainings and continuing education as working hours |  |
| (3) Full coverage of fees for trainings and continuing education |  |
| (4) Full coverage of travelling and accommodation costs for trainings and continuing education |  |
| (5) Offering inhouse and nearby trainings and continuing education |  |
| Organizational strategies: Occupational health promotion | |
| Our organization enables measures for occupational health promotion for staff (e.g. cooperation with gyms, courses on stress management). | 1=do not agree at all, 2=do not agree, 3=agree, 4=completely agree |
| Organizational strategies: Supervision for staff | |
| In our organization supervision for the staff is carried out. | 1=do not agree at all, 2=do not agree, 3=agree, 4=completely agree |
| Organizational strategies: Quality management | |
| Our organization has a uniform quality management. | 1=do not agree at all, 2=do not agree, 3=agree, 4=completely agree |
| Organizational strategies: External staff | |
| Has external staff (honorary staff, temporary employees) been employed in your organization within the last 12 months? | 0=no, 1=yes |
| Organizational strategies: Outsourcing | |
| Have parts of your organization been outsourced? | 0=no, 1=yes |
